# Evaluation of [^18^F]F-DPA as a target for TSPO in head and neck cancer under normal conditions and after radiotherapy

**DOI:** 10.1007/s00259-020-05115-z

**Published:** 2020-12-19

**Authors:** Sanni Tuominen, Thomas Keller, Nataliia Petruk, Francisco López-Picón, Dominik Eichin, Eliisa Löyttyniemi, Alejandra Verhassel, Johan Rajander, Jouko Sandholm, Johanna Tuomela, Tove J. Grönroos

**Affiliations:** 1 Preclinical Imaging Laboratory, Turku PET Centre, University of Turku, Tykistökatu 6A, FI-20520 Turku, Finland; 2 Institute of Biomedicine and FICAN West Cancer Research Laboratory, University of Turku, Kiinamyllynkatu 10, FI-20520 Turku, Finland; 3 MediCity Research Laboratory, University of Turku, Tykistökatu 6A, FI-20520 Turku, Finland; 4 Radiopharmaceutical Chemistry Laboratory, Turku PET Centre, University of Turku, Kiinamyllynkatu 4-8, FI-20520 Turku, Finland; 5 Department of Biostatistics, University of Turku, Kiinamyllynkatu 10, FI-20520 Turku, Finland; 6 Accelerator Laboratory, Turku PET Centre, Åbo Akademi University, Kiinamyllynkatu 4-8, FI-20520 Turku, Finland; 7 Turku Bioscience Centre, University of Turku and Åbo Akademi University, Tykistökatu 6A, FI-20520 Turku, Finland; 8 Department of Oncology and Radiotherapy, Turku University Hospital, Hämeenkatu 11, FI-20520 Turku, Finland

**Keywords:** [^18^F]F-DPA, TSPO, PET, Radiotherapy, Head and neck cancer

## Abstract

**Background:**

Many malignant tumours have increased TSPO expression, which has been related to a poor prognosis. TSPO-PET tracers have not comprehensively been evaluated in peripherally located tumours. This study aimed to evaluate whether N,N-diethyl-2-(2-(4-([^18^F]fluoro)phenyl)-5,7-dimethylpyrazolo[1,5-a]pyrimidin-3-yl)acetamide ([^18^F]F-DPA) can reflect radiotherapy (RT)-induced changes in TSPO activity in head and neck squamous cell carcinoma (HNSCC).

**Methods:**

RT was used to induce inflammatory responses in HNSCC xenografts and cells. [^18^F]F-DPA uptake was measured in vivo in non-irradiated and irradiated tumours, followed by ex vivo biodistribution, autoradiography, and radiometabolite analysis. In vitro studies were performed in parental and TSPO-silenced (TSPO siRNA) cells. TSPO protein and mRNA expression, as well as tumour-associated macrophages (TAMs), were also assessed.

**Results:**

In vivo imaging and ex vivo measurement revealed significantly higher [^18^F]F-DPA uptake in irradiated, compared to non-irradiated tumours. In vitro labelling studies with cells confirmed this finding, whereas no effect of RT on [^18^F]F-DPA uptake was detected in TSPO siRNA cells. Radiometabolite analysis showed that the amount of unchanged [^18^F]F-DPA in tumours was 95%, also after irradiation. PK11195 pre-treatment reduced the tumour-to-blood ratio of [^18^F]F-DPA by 73% in xenografts and by 88% in cells. TSPO protein and mRNA levels increased after RT, but were highly variable. The proportion of M1/M2 TAMs decreased after RT, whereas the proportion of monocytes and migratory monocytes/macrophages increased.

**Conclusions:**

[^18^F]F-DPA can detect changes in TSPO expression levels after RT in HNSCC, which does not seem to reflect inflammation. Further studies are however needed to clarify the physiological mechanisms regulated by TSPO after RT.

**Supplementary Information:**

The online version contains supplementary material available at 10.1007/s00259-020-05115-z.

## Introduction

Translocator protein (TSPO)-targeting positron emission tomography (PET) imaging is currently mainly used for imaging neurodegenerative diseases [1] and brain gliomas [2] as TSPO is considered a biomarker of neuroinflammation and microglial activation. Many cancer types exhibit increased TSPO expression, which has been related to an aggressive phenotype and/or poor prognosis [3, 4]. Imaging of TSPO in cancer could therefore be a useful tool in treatment planning and/or for the development of new TSPO-targeting drugs. Apart from brain gliomas, only a few preclinical studies have evaluated TSPO-PET tracers in peripherally located tumours [5–9]. Many of these reports focused on evaluating the relationship between tracer uptake and tumour inflammation and/or levels of macrophages [5–8]. Several studies [5, 6, 8] have reported that TSPO imaging has potential in measuring macrophage levels in tumours, whereas Zheng et al. [7] reported on a low lesion-to-background uptake with [^18^F]DPA-714 in several models for cancer and inflammation. Tantawy et al. [9] concluded that [^18^F]VUIIS1008 might be a useful tracer for TSPO-targeting in prostate cancer.

TSPO, located on the outer mitochondrial membrane, has been suggested to participate in numerous cellular processes, including steroid biosynthesis, cholesterol transport, apoptosis, cell proliferation, immune response, mitochondrial metabolism, and oxidative stress [10]. Originally, the main role of TSPO was thought to mediate mitochondrial cholesterol import for steroid hormone production. Recent discoveries that global deletion of TSPO in mice does not affect viability, fertility, or the ability to generate steroid hormones [11–13], have challenged our understanding of the physiological function of TSPO. Furthermore, the role of TSPO in regulating mitochondrial membrane permeability and apoptosis has been challenged by recent studies [14–17], whereas growing evidence supports a regulatory role of TSPO in mitochondrial energy [18, 19] and reactive oxygen species (ROS) [20–22] homeostasis. It has been speculated that the pathological meaning of altered TSPO binding or expression are disease-specific, and therefore not easily generalizable across different neuropathologies or inflammatory conditions [19, 23].

Thus, the aim of this study was to comprehensively evaluate the TSPO tracer, N,N-diethyl-2-(2-(4-([^18^F]fluoro)phenyl)-5,7-dimethylpyrazolo[1,5-a]pyrimidin-3-yl)acetamide ([^18^F]F-DPA) [24], in head and neck squamous cell carcinoma (HNSCC). The main difference between [^18^F]F-DPA (Ki = 1.7 nM) and another TSPO-PET tracer in use, [^18^F]DPA-714 (Ki = 7.0 nM), is that the fluorine atom is directly linked to the phenyl moiety in [^18^F]F-DPA, whereas the presence of an alkyl or alkoxy spacer chain is needed in [^18^F]DPA-714. The binding of the fluorine-18 atom directly to the aromatic ring is more favourable with respect to in vivo radioligand metabolism and [^18^F]fluoride release.

In order to induce inflammatory conditions, xenografts and cells were irradiated and the effect of radiotherapy (RT) on the [^18^F]F-DPA uptake was evaluated. The uptake of [^18^F]F-DPA was determined after blocking TSPO with PK11195 and in TSPO silenced (TSPO siRNA) cells and the expression of TSPO mRNA and protein levels were measured. The metabolic profile and biodistribution of [^18^F]F-DPA was also determined. Finally, the effect of RT on tumour-associated macrophages (TAMs) was measured in order to evaluate their impact on the tracer uptake in tumours.

## Materials and methods

### Radiosynthesis

[^18^F]FDG was synthesized at the Radiopharmaceutical Chemistry Laboratory of Turku PET Centre using the FASTlab synthesizer (GE Healthcare) as described previously [25]. Radiochemical purity exceeded 98% in all syntheses, and the molar activity (A_m_) at the end of the synthesis (EOS) was > 100 GBq/μmol.

[^18^F]F-DPA was synthesized via two different approaches resulting in different A_m_s. High A_m_ (360–900 GBq/μmol at EOS) [^18^F]F-DPA was produced by a copper-mediated nucleophilic ^18^F-fluorination methodology [26]. The electrophilic syntheses of [^18^F]F-DPA, resulting in lower A_m_ (10 GBq/μmol at EOS), were performed according to previously described procedures [24].

### Cell culture

FaDu cells (ATCC Cat# HTB-43, RRID:CVCL_1218) were cultured in DMEM supplemented with 10% heat-inactivated Fetal Bovine Serum (FBS), E.U. Approved (Country of Origin: Brazil; Source: Cattle/Bovine), L-glutamine, MEM NEAA, and penicillin-streptomycin. Cal33 cells were a kind gift from Prof. Anna Dubrovska (OncoRay–National Center for Radiation Research in Oncology, Medizinische Fakultät Dresden, Germany). Cells were cultured in DMEM supplemented with 10% heat-inactivated FBS, L-glutamine, HEPES, MEM NEAA, sodium pyruvate, and penicillin-streptomycin. All reagents were purchased from Gibco Thermo Fisher. FaDu cells were identified by Short Tandem Repeat (STR) profiling, and Cal33 cells were genotyped using microsatellite polymorphism analysis.

### TSPO silencing in cells

Parental FaDu cells were silenced with TSPO (TSPO siRNA) or non-targeting (NT) siRNA according to the manufacturer’s protocol (Accell siRNA, Dharmacon Horizon Discovery, https://horizondiscovery.com/en/products/gene-modulation/knockdown-reagents/sirna/PIFs/Accell-siRNA-Reagents-Human#resources; PDF files can be found in the “Resources” tab). Briefly, 1 × 10^4^ cells were seeded in 96-well plates and NT or TSPO siRNA was added at a concentration of 1 μM for 72 h.

### Xenografts

**Fig. 1:**
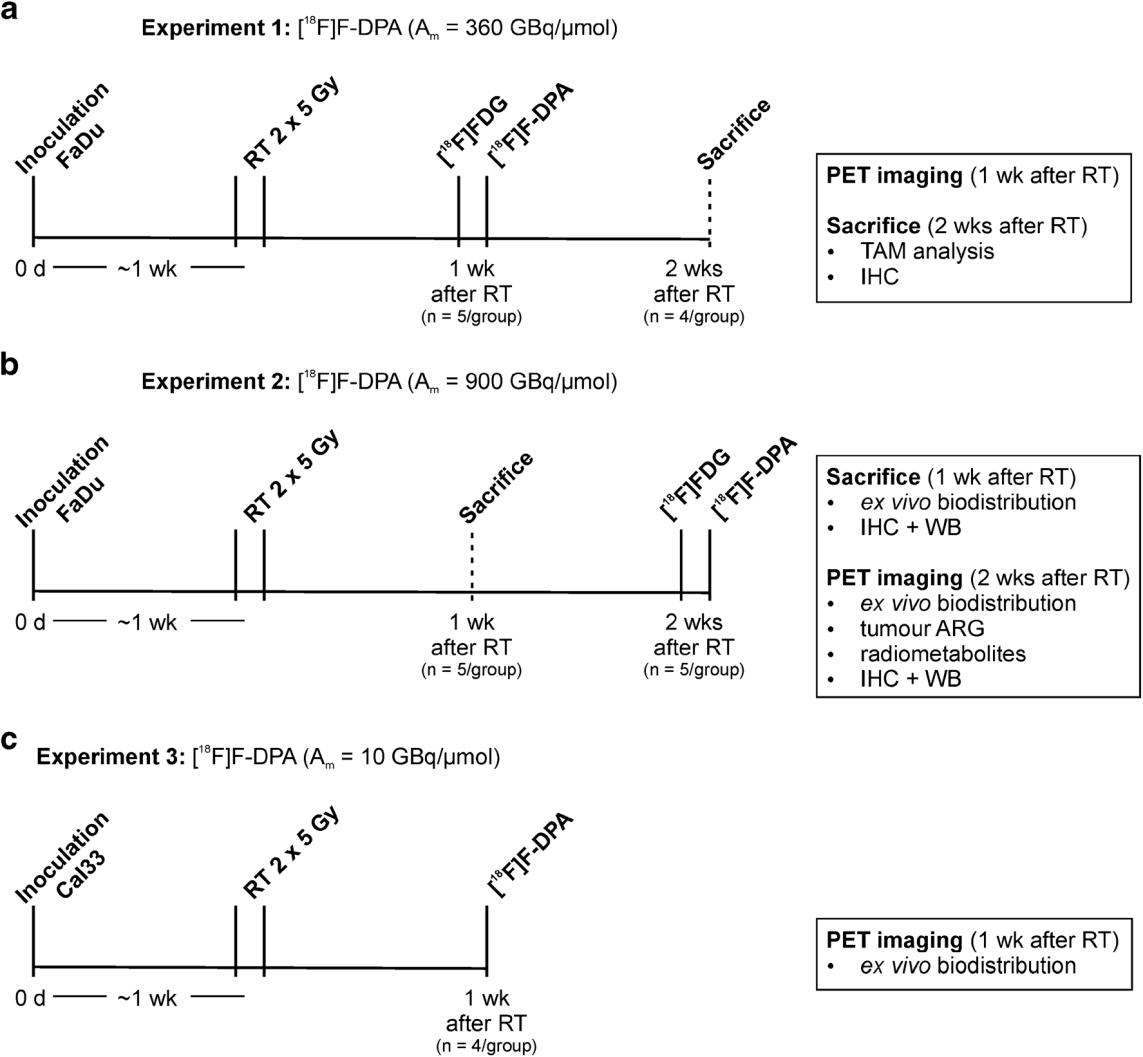
Time schedule and set-up of the three in vivo experiments performed in this study. [^18^F]FDG and [^18^F]F-DPA imaging of FaDu tumour bearing mice was performed on consecutive days **a** 1 or **b** 2 weeks (wk) after radiotherapy (RT). **c** [^18^F]F-DPA imaging of Cal33 tumour bearing mice was performed 1 week after RT. *TAM* tumour-associated macrophages, *IHC* immunohistochemistry, *WB* Western blot, *ARG* autoradiography

In total, 34 female nude mice (Hsd;athymic Nude-Foxn1^nu^, age 4–6 weeks, Envigo) were housed under controlled pathogen-free environmental conditions. Animals were cared for in accordance with Directives 2012/707/EU and 2014/11/EU and the European Parliament and Council for the Care and Use of Laboratory Animals. Ethical approval (license No: ESAVI/2329/04.10.07/2017) of the study was obtained from the ethics committee (Regional State Administrative Agencies in Finland). Cells (1 × 10^6^) were inoculated subcutaneously into the left or right hind limb. When the tumour diameter was 5–6 mm [27], mice were stratified into non-irradiated (Ctrl) and irradiated (RT) groups. The experimental set-ups, number of animals, injected doses, and A_m_s of [^18^F]F-DPA, used for each experiment, are presented in Fig. 1 and Table 1.

**Table 1:** The total number of tumour bearing mice and amount of injected doses used in the study

	FaDu ^a^ (Experiment 1)					FaDu ^b^ (Experiment 2)				Cal33 ^c^ (Experiment 3)			
	1 week after RT					2 weeks after RT				1 week after RT			
	Ctrl	RT	Pretrt.	Injected dose	Injected mass	Ctrl	RT	Injected dose	Injected mass	Ctrl	RT	Injected dose	Injected mass
	(n)	(n)	(n)	(MBq)	(ng)	(n)	(n)	(MBq)	(ng)	(n)	(n)	(MBq)	(ng)
PET imaging				Mean ± SD	Mean ± SD			Mean ± SD	Mean ± SD			Mean ± SD	Mean ± SD
[^18^F]FDG	5	5		4.97 ± 0.59	N/A	6	5	5.14 ± 0.30	N/A				
[^18^F]F-DPA	5	5	5	2.95 ± 1.03	15.5 ± 3.69	5	5	4.05 ± 0.77	13.3 ± 3.25	3	3	2.10 ± 0.13	664 ± 233
Ex vivo ([^18^F]F-DPA)													
Biodistribution: whole body	3	3		3.61 ± 0.79	606 ± 311								
Biodistribution: selected tissues	9	10		3.15 ± 1.07	352 ± 372*					4	4	2.09 ± 0.11	648 ± 276
Tumour autoradiography													
Ex vivo	2	2	2	3.99 ± 0.67	797 ± 123								
In vitro	2	2	2	see text**	see text**								
Radiometabolite analyses	2	2	2	3.99 ± 0.67	797 ± 123								
TAM analyses						4	4						
IHC	4	4				11	5						
Western blot	4	4				5	4						

Animal weights (mean ± SD) in ^a^ 22.9 ± 1.31, ^b^ 21.9 ± 0.90 and ^c^ 16.9 ± 0.81

*Syntheses with both higher and lower A_m_s were used

**The amount of added tracer is described in the materials and methods section, “in vitro autoradiography”

### Irradiation of xenografts and cells

Xenograft bearing mice were locally irradiated (Faxitron MultiRad 350) to tumour area only under isoflurane anaesthesia with 5 Gy on two consecutive days (320 kV X-rays, 10 mA current, SnCuAl filter, source of surface distance 45.0 cm, 1 Gy/min, total dose 10 Gy). Parental FaDu (5 × 10^5^), NT siRNA (1 × 10^5^) and TSPO siRNA (1 × 10^5^) cells were allowed to attach to 6-well plates overnight, following irradiations with a 2 Gy dose on five consecutive days (320 kV X-rays, 10 mA current, Al 0.5 mm filter, source of surface distance 37.0 cm, 6 Gy/min, total dose 10 Gy).

### PET/CT imaging and data analysis

PET/CT imaging was done with mice 1 or 2 weeks after RT-treatment (Fig. 1). Imaging of FaDu tumours (Inveon, Siemens) was performed with [^18^F]FDG and [^18^F]F-DPA on two consecutive days, whereas Cal33 tumours were only imaged (x- and β-cubes, Molecubes) with [^18^F]F-DPA.

Tracers were injected intravenously *via* a cannula inserted into the tail vein under isoflurane anaesthesia. A 20-min static [^18^F]F-DPA scan was started 20 min after injection. The scanning time point was chosen based on dynamic imaging data. Mice imaged with [^18^F]FDG were kept anesthetized for 60 min before starting a 20-min static scan. In order to block TSPO, pre-treatment with PK11195 (1 mg, i.p admin., 30 min prior to [^18^F]F-DPA injection, Sigma) was done in non-irradiated mice. Imaging data was collected in list mode and reconstructed with an OSEM3D algorithm. ^18^F-radioactivity uptake in tumours was calculated as a percentage of injected dose per millilitre of tissue (% ID/mL). Values were corrected for the injected activity and decay.

### Radiometabolite analyses

The metabolite analyses were performed with non-irradiated, irradiated and PK11195 pre-treated FaDu tumours 40 min after [^18^F]F-DPA injection. Plasma proteins were precipitated by adding 1.5 parts (volume) of methanol. Tumour samples were homogenized with approximately 200 μL of 1:1 (v/v) methanol and water. After centrifugation (12 000 × g, 4 min), 30 μL of supernatant was spotted onto an aluminum-backed silica gel 60/Kieselguhr F_254_ TLC plate (Merck Millipore, art. no 1.05567). The thin-layer chromatographic (TLC) analysis was carried out according to previously published methods [28]. The proportion (%) of intact tracer in total ^18^F-radioactivity of samples was calculated.

### Ex vivo tissue counting and autoradiography

[^18^F]F-DPA was allowed to accumulate for 40 min before the mice were sacrificed by cardiac puncture and blood and tissues were dissected, weighed, and measured for ^18^F-radioactivity with a gamma counter (2480 WIZARD2, PerkinElmer). Measured radioactivity was corrected for decay and background and expressed as percentage of injected dose per gram of tissue (% ID/g tissue) or as muscle-to-blood (M/B) and tumour-to-blood (T/B) ratios. After measuring, FaDu tumours were frozen in chilled isopentane and cut into 20-μm sections using a cryomicrotome (Microm HM 500 OM) and exposed to an imaging plate and scanned with the Fuji Analyzer BAS5000 (resolution 25 μm).

### In vitro autoradiography

Frozen non-irradiated and irradiated FaDu tumour sections (20 μm) were first pre-incubated in Tris-NaCl for 5 min (+ 4 °C) and then incubated with [^18^F]F-DPA (2 nmol/L) for 1 h (room temperature). For blocking studies, PK11195 (1000 nmol/L) was co-incubated with [^18^F]F-DPA. Brain sections from a 17-month-old APP/PS1-21 transgenic mouse (model of Alzheimer’s disease) were used as positive controls under the same conditions [28]. Slides were washed twice with Tris-NaCl and once with dH_2_O (5 min, + 4 °C), exposed to an imaging plate and scanned with the Fuji Analyzer BAS5000.

### Proportion of monocytes and tumour-associated macrophages

Tumours were minced and digested in 3 mL RPMI with 2% Hepes, 2% FCS, 0.5 mg/mL Collagenase D and 0.1 mg/mL DNAse 1 at + 37 °C on a shaker (enzymes from Roche). After 45 min 300 μL 0.1 M EDTA was added for 5 min to stop the reaction. Single cell suspension was then obtained by using gentleMACS C tubes with a gentleMACS Dissociator and filtering of the suspension. Cells were blocked with BD’s FC-block (Cat# 553141) 30 min on ice, stained with directly conjugated antibodies from BD (30 min on ice) and a viability dye, recorded on a LSR Fortessa flow-cytometer (BD, BioSciences) and analysed with Flowjo v10 (FlowJo LLC). The used antibodies and dyes were: Fixable Viability Dye eFluor 780 (eBioscience, Cat# 65-0865-14), CD11b FITC (BioLegend, Cat# 101206), Ly6G PerCP-Cy5.5 (BD, Cat# 560602), F4/80 APC (eBiosicence, Cat# 17-4801-82), MHCII PE (BD, Cat# 557000), Ly6C-BV421 (BD, Cat# 562727).

### Immunohistochemical staining

The following antibodies were used for staining: TSPO (1:5,000; Abcam Cat# ab109497), cleaved caspase-3 (Cas-3; 1:500; Cell Signaling Technology Cat# 9664, RRID:AB_2070042) and phospho-histone H3 (PHH3; 1:100; Cell Signaling Technology Cat# 9701, RRID:AB_331535). The percentages of cells from ROIs with a 700-μm radius staining positively for PHH3 and Cas-3 were analysed. For TSPO, positively stained cells from the whole tumour area were analysed. All the analyses were done with QuPath [29]. The analysis scripts are shown in Supplementary Tables 1 and 2.

### In vitro uptake of [^18^F]F-DPA in FaDu cells

The uptake of [^18^F]F-DPA in parental FaDu, FaDu NT and TSPO siRNA cells was measured approximately 5 h after the last irradiation. Cells were incubated with 0.5 MBq/mL [^18^F]F-DPA for 60 min. The number of samples, tracer concentrations and added masses are presented in Table 2. In order to block TSPO cells were incubated with PK11195 (10 μM) for 30 min prior to tracer administration where after the media was replaced with a solution containing 0.5 MBq/mL of [^18^F]F-DPA and 10 μM PK11195. The PK11195 concentration was selected based on experiments with different concentrations (Supplementary Fig. 1). After incubation, the cells were washed twice with PBS and detached with Trypsin-EDTA. Trypsin-EDTA was inactivated with 1:1 (vol/vol) heat-inactivated FBS and PBS and the cells were collected to Eppendorf tubes. The number of viable cells was counted (Cellometer auto 2000, Nexcelom) and the ^18^F-radioactivity measured by a gamma counter (2480 Wizard2, PerkinElmer).

**Table 2:** The total number of experiments used in the in vitro study

	FaDu					FaDu TSPO siRNA		FaDu NT siRNA			
	Ctrl	RT	Pretrt.	Dose	Mass	Ctrl	RT	Ctrl	RT	Dose	Mass
	(n)	(n)	(n)	(MBq/mL)	(ng/mL)	(n)	(n)	(n)	(n)	(MBq/mL)	(ng/mL)
				Mean ± SD	Mean ± SD					Mean ± SD	Mean ± SD
[^18^F]F-DPA labelling	4 *(14)*	4 *(14)*	1 *(3)*	0.43 ± 0.12	37.4 ± 41.4 *	2 *(7)*	2 *(7)*	2 *(7)*	2 *(7)*	0.39 ± 0.12	40.2 ± 16.0
Western blot	4 *(13)*	4 *(13)*				2 *(7)*	2 *(7)*	2 *(7)*	2 *(7)*		
qPCR	4 *(11)*	4 *(10)*				2 *(7)*	2 *(7)*	2 *(7)*	2 *(7)*		

Numbers shown in italic refers to the total amount of parallel wells used in the experiments

*Syntheses with both higher and lower A_m_s were used

### Western blot analysis

Tumour samples and cells were lysed in RIPA buffer containing phosphatase and protease inhibitors (Thermo Scientific). The protein concentration was measured using the bicinchoninic acid method (Pierce BCA Protein Assay Kit, Thermo Scientific) and equal amounts of protein (30 μg) were loaded and electrophoresed in 4–20% gradient gels (Mini-PROTEAN TGX Precast Protein Gels, Bio-Rad). Samples were transferred to a nitrocellulose membrane and the membranes incubated overnight (+ 4 °C) with the following primary antibodies: TSPO (1:1,000; Abcam Cat# ab109497, RRID:AB_10862345), γH2A.x (1:500; Abcam Cat# ab22551, RRID:AB_447150) and GAPDH (1:10,000; Abcam Car# ab181602; RRID not available). γH2Ax protein expression functions as a sensitive marker of RT-induced DNA double-strand breaks [30]. Fluorescent secondary antibody (1:2,000 in TBS-T, IRDye 800CW LI-COR Biosciences Cat# 926-32213, RRID:AB_621848 or IRDye 680 RD Secondary Antibody, LI-COR Biosciences Cat# 926-68072, RRID:AB_10953628) was added for 1 h at room temperature. Protein bands were detected with LI-COR Odyssey CLx.

### Real-time quantitative PCR

Cell samples were lysed in 1:1 (v/v) RLT buffer (Qiagen) and 96% ethanol. Total RNA was isolated using the RNeasy Plus Mini Kit (Qiagen) according to the manufacturer’s instructions. RNA was converted into cDNA using Oligo d(T) 18 mRNA Primer (New England BioLabs), dNTP Mix, RiboLock RNAse Inhibitor, RT Buffer, and Maxima Reverse Transcriptase (all from Thermo Scientific). For real-time quantitative PCR (RT-qPCR), 30 ng of cDNA was used with SsoAdvanced Universal SYBR Green Supermix (Bio-Rad). The primers were hTSPO (Bio-Rad, sequence not available), hTBP [31], and hRPLP0 [32]. Data analysis was done from C_t_ values normalized to the average value of two housekeeping genes (TBP and RPLP0). Results are shown as fold-change expression to Ctrl (delta-delta C_t_ method).

### Statistical analysis

Results are given as mean ± standard deviation (SD). Statistical analyses were performed with Student’s *t* test or with one- or two-way analysis of variance, including at least group (cell line) and treatment (RT, Ctrl, Pre-treatment) and if needed time (1 or 2 weeks after RT) as explanatory variables and depending on the analysis a different variable (i.e. tracer uptake, T/B ratio, protein- or mRNA expression). Normality assumption was checked using studentized residuals. *p* values less than 0.05 (two-tailed) were considered statistically significant. The used software was SAS System, version 9.4 for Windows (SAS Institute Inc.).

## Results

### In vivo tumour uptake of [^18^F]F-DPA and [^18^F]FDG

**Fig. 2:**
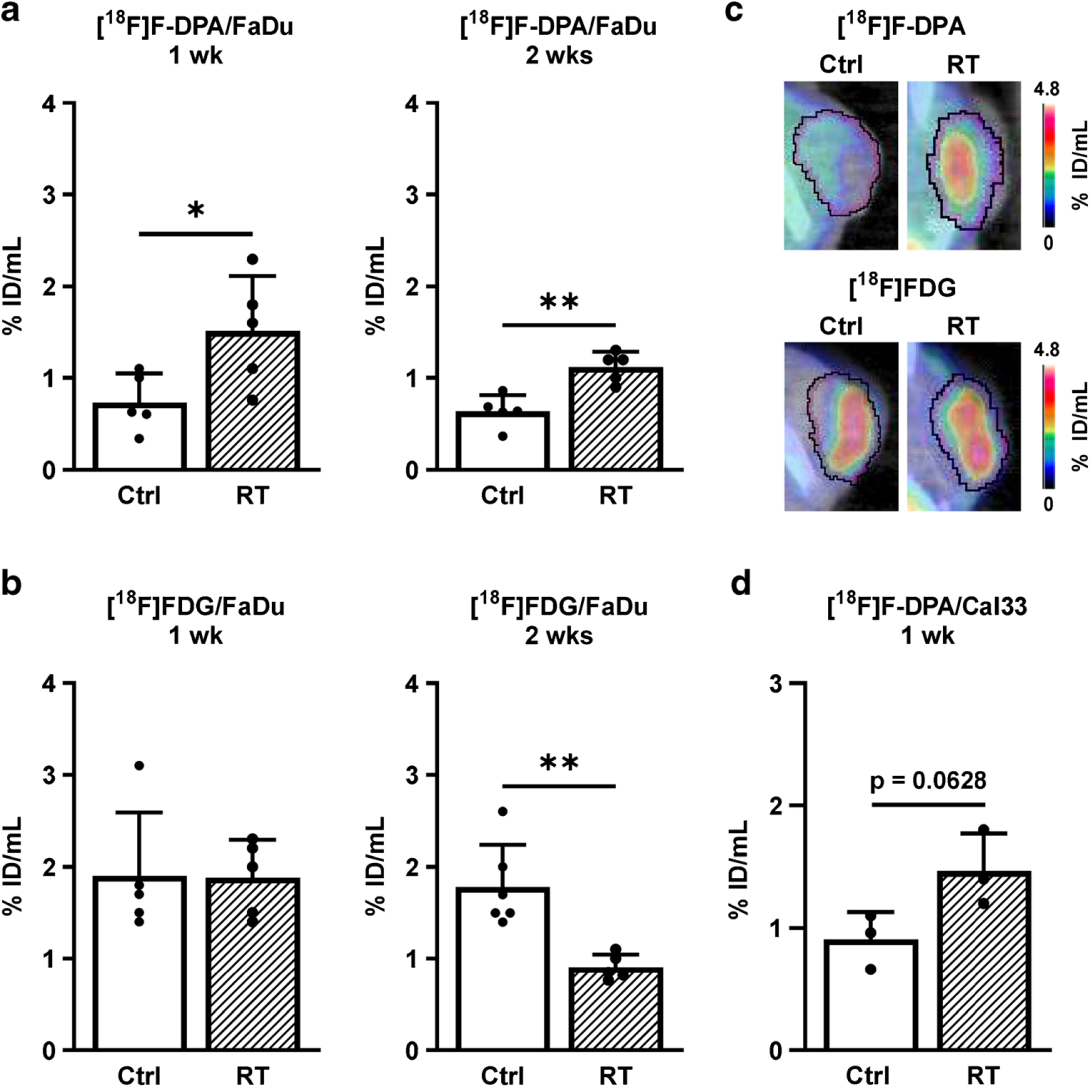
**a** In vivo uptake of [^18^F]F-DPA (summed 20–40 min post injection) in non-irradiated (Ctrl) and irradiated (RT, 2 × 5 Gy) FaDu tumours 1 and 2 weeks (wk) after RT. **b** The [^18^F]FDG uptake (summed 60–80 min post injection) was determined in same tumours on the previous day. **c** PET/CT images of [^18^F]F-DPA and [^18^F]FDG uptake in non-irradiated and irradiated FaDu tumours 1 week after treatment. **d** The uptake of [^18^F]F-DPA in Cal33 tumours 1 week after RT. Data is expressed as percentage of injected dose per millilitre tissue (% ID/mL) in whole tumours, mean ± SD, *n* = 3–6/group. Each data point represents one tumour. *p < 0.05, **p < 0.01 are considered to be statistically significant compared to controls, by two-tailed Student’s *t* test

Irradiation clearly decreased the tumour volume in all experiments (Supplementary Fig. 2). There were no changes in the weights (mean ± SD) of the mice in non-irradiated and irradiated groups in any experimental set-up. A significantly higher (*p* = 0.0329) [^18^F]F-DPA uptake was seen in irradiated tumours 1 week after RT (1.51 ± 0.60% ID/mL) compared to non-irradiated tumours (0.74 ± 0.31% ID/mL). A similar change was also seen 2 weeks after treatment (1.12 ± 0.16% ID/mL vs. 0.64 ± 0.18% ID/mL) in irradiated and non-irradiated tumours, respectively (*p* = 0.0024, Ctrl vs. RT) (Fig. 2a). The [^18^F]FDG uptake, measured in the same tumours, remained unchanged 1 week after RT (1.88 ± 0.41% ID/mL) compared to non-irradiated tumours (1.90 ± 0.69% ID/mL), whereas a significantly lower (*p* = 0.0025) uptake was detected after RT (0.91 ± 0.14% ID/mL) compared to non-irradiated tumours (1.78 ± 0.45% ID/mL) 2 weeks after treatment (Fig. 2b). Representative images of [^18^F]F-DPA and [^18^F]FDG uptake in the tumours 1 week after RT are shown in Fig. 2c.

In order to confirm our finding that RT increases [^18^F]F-DPA uptake, we also measured the uptake in Cal33 tumours 1 week after RT (Fig. 2d). Even though significance was not reached, a clear trend (*p* = 0.0628) towards a higher uptake was found after RT compared to non-irradiated tumours (1.47 ± 0.31 vs. 0.91 ± 0.22% ID/mL, respectively).

**Fig. 3:**
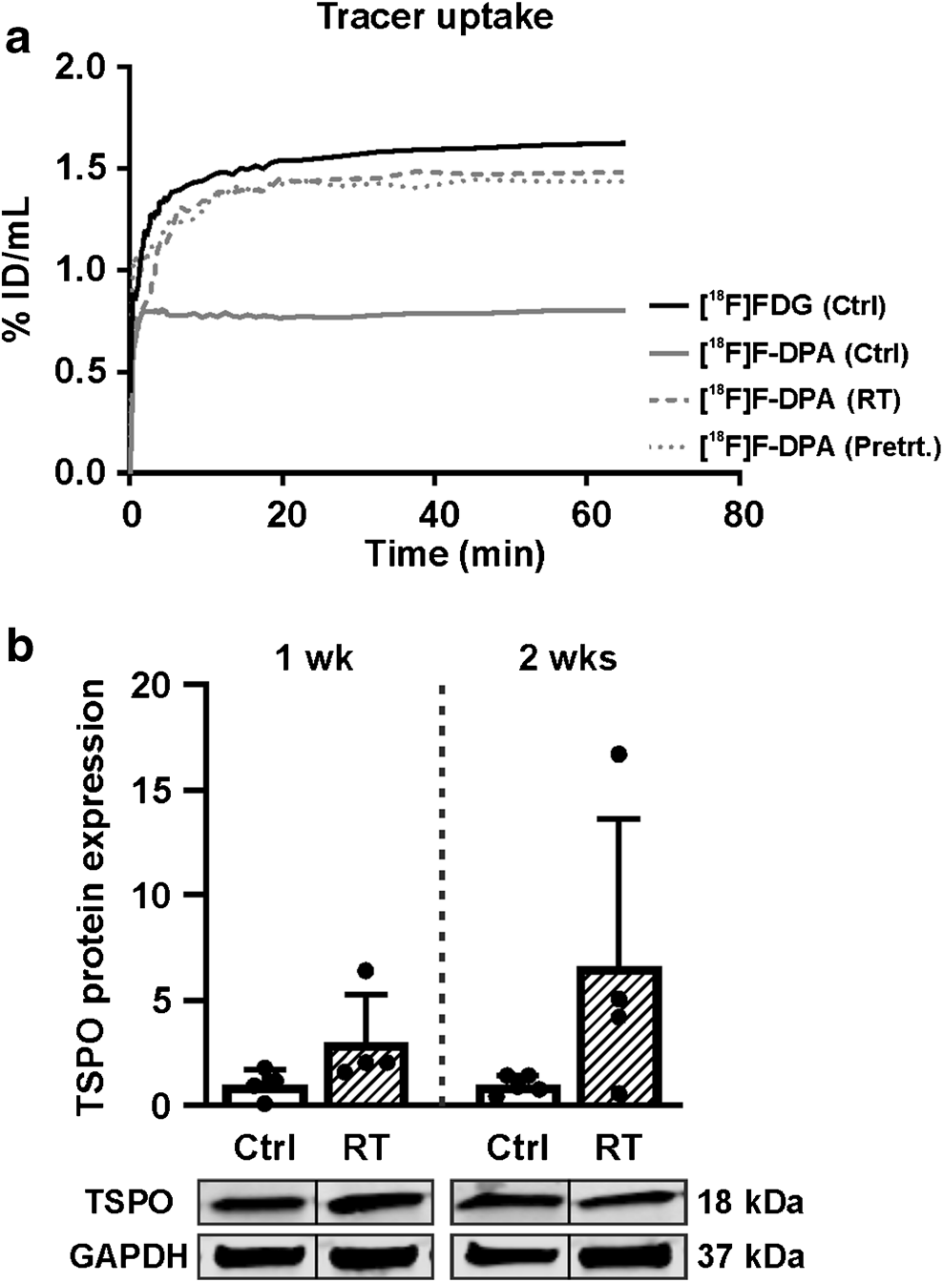
**a** Time-activity curves (TACs) derived from volumes of interests drawn over whole tumours after injection of [^18^F]FDG and [^18^F]F-DPA. The [^18^F]FDG TAC is shown from a non-irradiated (Ctrl) tumour, whereas the [^18^F]F-DPA TACs are shown for non-irradiated, irradiated (RT, 2 × 5 Gy), and pre-treated (Pretrt., 1 mg PK11195) tumours. **b** Expression of TSPO protein in non-irradiated and irradiated FaDu tumours 1 and 2 weeks (wk) after treatment. Data is expressed as relative protein expression normalized to housekeeping gene GAPDH, mean ± SD, *n* = 4–5/group. Each data point represents one tumour. *TSPO* Translocator protein, *GAPDH* Glyceraldehyde 3-phosphate dehydrogenase

Dynamic imaging data revealed an increased tumour uptake of [^18^F]F-DPA in both irradiated and PK11195 pre-treated groups compared to the non-irradiated tumour (Fig. 3a). [^18^F]F-DPA uptake in non-irradiated tumour was quite low compared to that seen with [^18^F]FDG.

### TSPO protein expression in tumours

Compared to non-irradiated tumours, the TSPO protein expression varied greatly after RT (Fig. 3b). The mean expression was higher in irradiated tumours compared to non-irradiated tumours 1 and 2 weeks after treatment, even though significance (*p* = 0.1393 vs. *p* = 0.1094, respectively) was lacking.

### Ex vivo [^18^F]F-DPA biodistribution

**Fig. 4:**
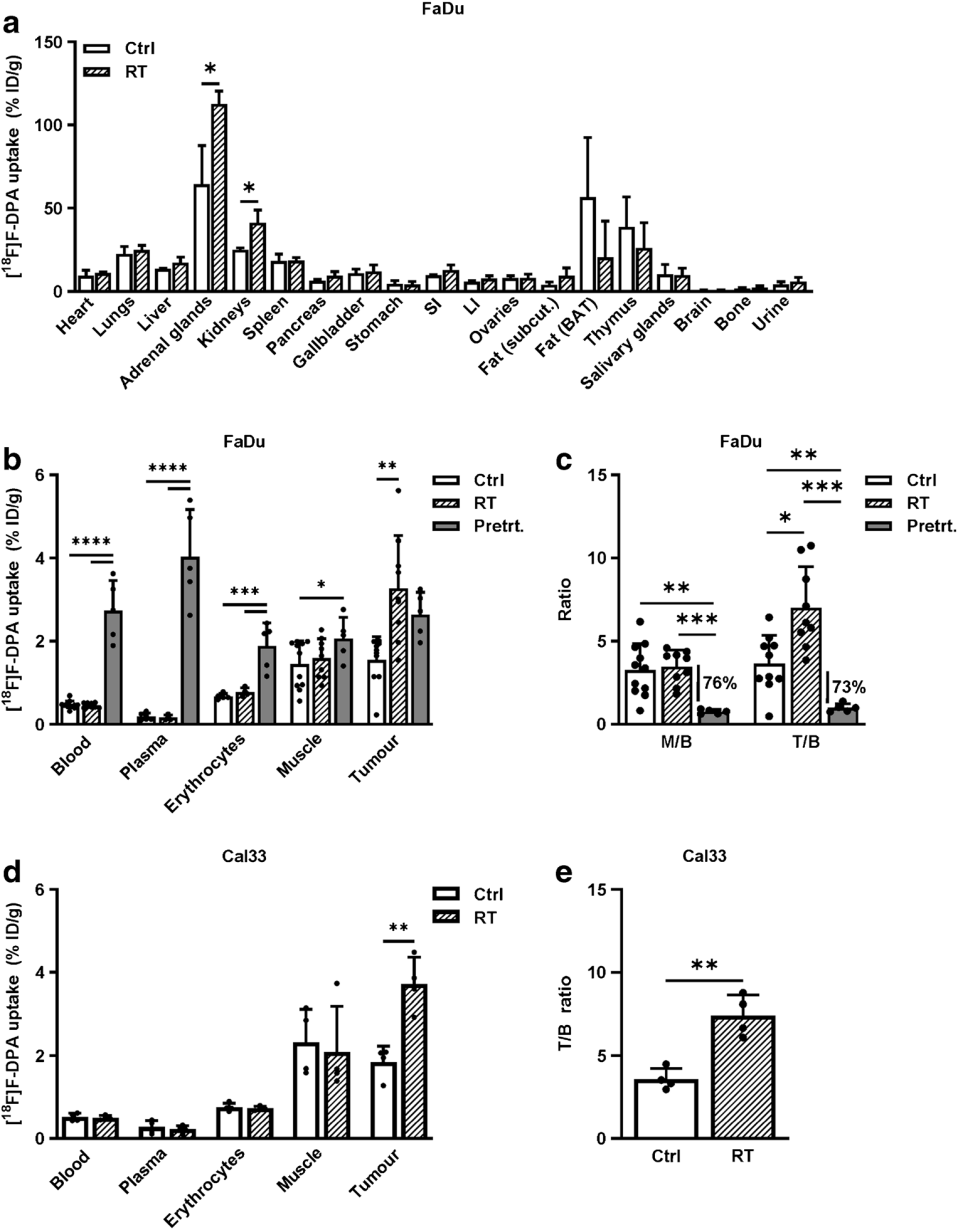
Ex vivo biodistribution of ^18^F-radioactivity in FaDu and Cal33 tumour bearing mice 40 min after [^18^F]F-DPA injection. **a** Whole body biodistribution in non-irradiated (Ctrl) and FaDu tumour bearing mice irradiated locally to tumour (RT, 2 × 5 Gy). Data is expressed as percentage of injected dose per gram tissue (% ID/g), mean ± SD, *n* = 3/group. *p < 0.05 is considered to be statistically significant compared to controls, by two-tailed Student’s *t* test. *SI* small intestine, *LI* large intestine, S*ubcut.* subcutaneous, *BAT* brown adipose tissue. **b**, **c** Uptake of [^18^F]F-DPA in blood components, muscle and tumour from non-irradiated, irradiated, and FaDu tumour bearing mice pre-treated with 1 mg of PK11195 30 min prior tracer injection. Data is expressed as percentage of injected dose per gram tissue (% ID/g), or as muscle-to-blood (M/B) and tumour-to-blood (T/B) ratios, mean ± SD, *n* = 5–10/group. *p < 0.05, **p < 0.01, ***p < 0.001, ****p < 0.0001 is considered to be statistically significant compared between each group, by one-way ANOVA. **d**, **e** Uptake of [^18^F]F-DPA in blood components, muscle and tumour from non-irradiated and irradiated Cal33 tumour bearing mice. Data is expressed as % ID/g, or as T/B ratios, mean ± SD, *n* = 4/group. **p < 0.01 is considered to be statistically significant compared to controls, by two-tailed Student’s *t* test

The whole body biodistribution of [^18^F]F-DPA (Fig. 4a and Supplementary Table 3) in mice, either given RT locally to tumours or non-irradiated, revealed that RT significantly increased the ^18^F-radioactivity accumulation in adrenal glands (*p* = 0.0277) and kidneys (*p* = 0.0225) compared to non-irradiated groups. In accordance with in vivo findings, the uptake of [^18^F]F-DPA was significantly (*p* = 0.0015) higher in irradiated (3.26 ± 1.01% ID/g) compared to non-irradiated (1.20 ± 0.70% ID/g) FaDu tumours (Fig. 4b). Other tissues and blood components remained unaffected by RT (Fig. 4a). Pre-treatment with PK11195 in non-irradiated mice significantly increased radioactivity uptake in blood (*p* < 0.0001), plasma (*p* < 0.0001), erythrocytes (*p* = 0.0002), and muscle (*p* = 0.0393) compared to non-treated non-irradiated mice. A trend towards a higher [^18^F]F-DPA uptake was also seen in non-irradiated tumours (*p* = 0.0751) pre-treated with PK11195 compared to non-irradiated non-treated tumours. No changes (*p* = 0.9781) in the mean M/B ratios from non-irradiated (3.26 ± 1.59) and irradiated (3.46 ± 1.01) mice was detected (Fig. 4c). In non-irradiated PK11195 pre-treated animals the M/B ratio (0.77 ± 0.13) was significantly lower compared to muscle from non-irradiated (*p* = 0.0012) and irradiated (*p* = 0.0001) mice. The mean T/B ratio was significantly higher in irradiated tumours (7.40 ± 2.29, *p* = 0.0124) compared to non-irradiated (3.07 ± 2.08) tumours (Fig. 4c). In non-irradiated PK11195 pre-treated tumours the T/B ratio (1.00 ± 0.26) was significantly lower compared to non-irradiated (*p* = 0.0022) and irradiated (*p* = 0.0003) tumours. The uptake of [^18^F]F-DPA in Cal33 tumours is shown in Fig. 4d and 4e. Again, a significantly higher [^18^F]F-DPA uptake (*p* = 0.0025) and T/B ratio (*p* = 0.0038) was measured in irradiated tumours compared to non-irradiated tumours. One tumour was excluded from the irradiated FaDu group because it was contaminated with radioactivity from urine.

### Radiometabolite analyses

**Fig. 5:**
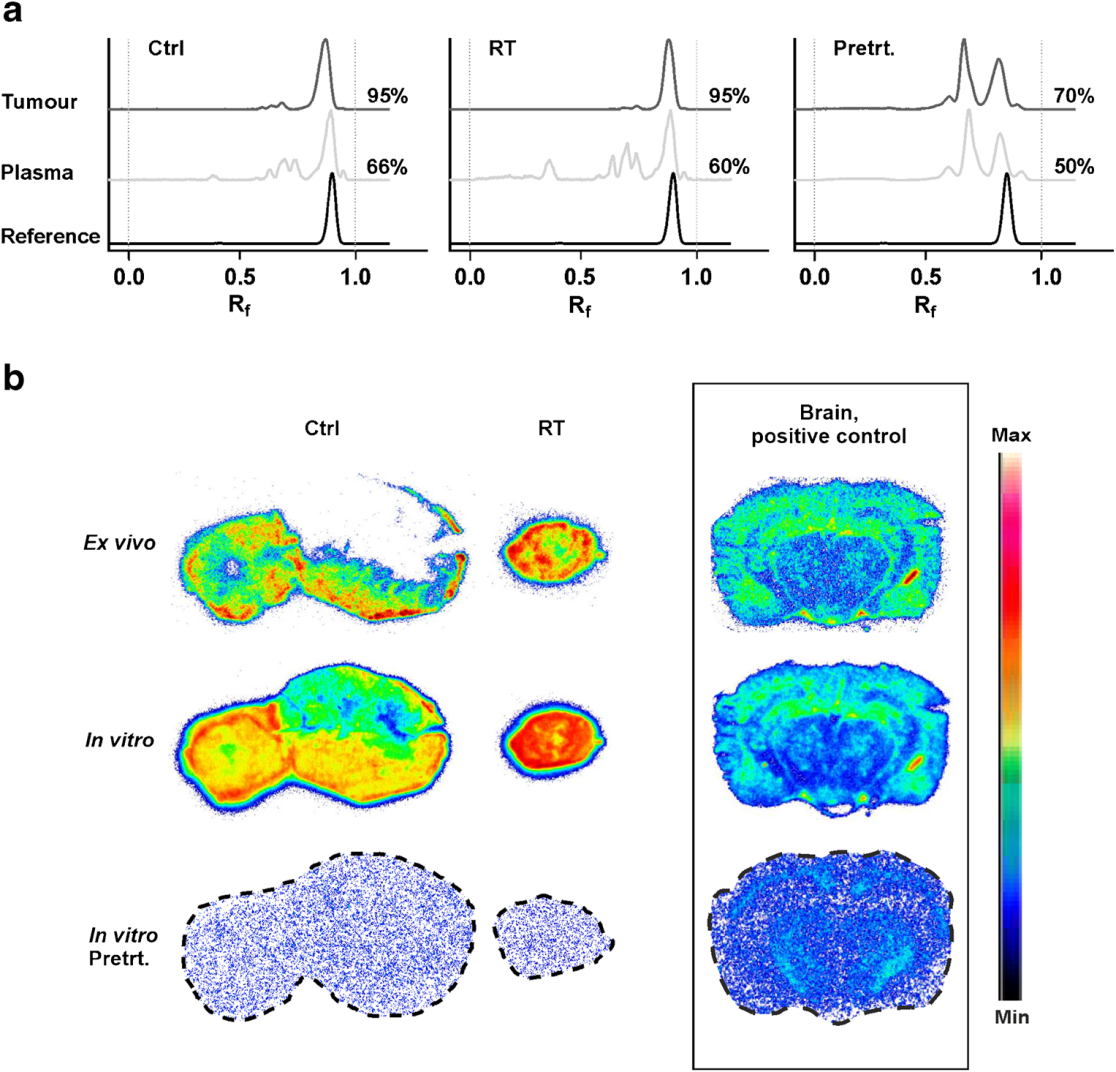
**a** RadioTLC chromatograms of plasma and tumour homogenates from non-irradiated (Ctrl), irradiated (RT, 2 × 5 Gy), and FaDu tumour bearing mice pre-treated (Pretrt.) with 1 mg PK11195 (i.p. injection) 30 min prior to tracer injection. Analyses were carried out 40 min after [^18^F]F-DPA administration 1 week after RT. Chromatogram of parental [^18^F]F-DPA included for reference. Values are average percentages of unchanged tracer from two independent experiments. *R*_*f*_ Retention value. **b** Ex vivo tumour autoradiography from non-irradiated and irradiated mice. Autoradiography was performed directly after in vivo imaging 1 week after RT (upper row). Sections incubated in vitro with [^18^F]F-DPA (2 nmol/L) for 1 h and pre-treated with PK11195 (1000 nmol/L) in vitro are shown in the middle and lower row, respectively. Brain sections from a 17-month-old APP/PS1-21 transgenic mouse (model of Alzheimer’s disease) were used as positive controls under same conditions

Five radiometabolites were visible in the plasma with R_f_ values of 0.50, 0.60, 0.65, 0.70, and 0.95 (Fig. 5a). These accounted for, on average, a total of 34%, 40% and 50% in plasma from non-irradiated, irradiated and PK11195 pre-treated mice, respectively, 40 min after injection of [^18^F]F-DPA. In tumours, the same radiometabolites were discovered, accounting for only approximately 5% of the remaining activity 40 min post injection. The mean unchanged tracer (R_f_ = 0.88) in non-irradiated and irradiated tumours accounted for 95%, whereas only 70% of the ^18^F-radioactivity represented unchanged tracer in tumours from pre-treated mice.

### Ex vivo and in vitro autoradiography

The ex vivo autoradiography images demonstrated much higher [^18^F]F-DPA uptake in irradiated and non-irradiated FaDu tumours compared to brain from an APP/PS1-21 transgenic mouse (Fig. 5b). Furthermore, images illustrate a higher tracer uptake in the irradiated tumour compared to the non-irradiated tumour. Negligible [^18^F]F-DPA uptake was detected in both non-irradiated and irradiated tumours after pre-treatment with PK11195 in vitro.

### Immunohistochemical analysis

Representative images and quantification of immunohistochemical staining against TSPO, Cas-3, and PHH3 are shown in Supplementary Fig. 3. The number of mitotic cells significantly decreased in irradiated tumours 1 week after RT compared to non-irradiated tumours. Otherwise, no significant changes in the amount of positive cells between the groups were detected.

### RT affects the proportion of monocytes and TAMs in FaDu xenografts

**Fig. 6:**
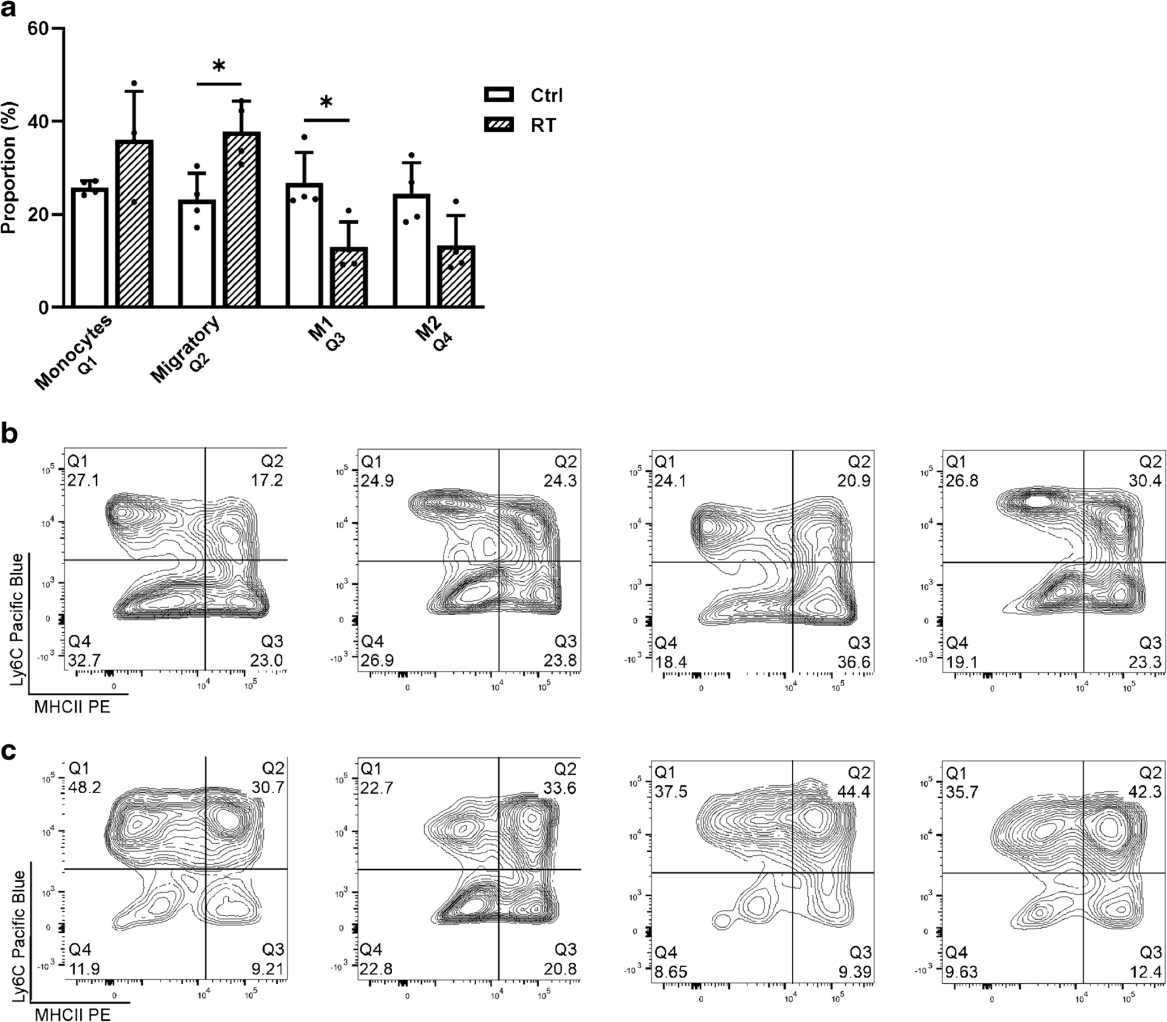
**a** Proportion of monocytes, immature/migratory macrophages and macrophages in stage M1 and M2 in non-irradiated and irradiated FaDu tumours. Data is expressed as percentage of total monocyte/macrophage population, mean ± SD, *n* = 4. *p < 0.05 is considered to be statistically significant compared to controls, by two-tailed Student’s *t* test. Flow cytometry charts depicting the population-proportions from **b** non-irradiated and **c** irradiated FaDu tumours. *Q1* monocytes, *Q2* immature/migratory macrophages, *Q3* M1 stage macrophages, *Q4* M2 stage macrophages. The gating strategy for selecting the subpopulations is shown in Supplementary Fig. 4

After RT, a slight increase in the number of monocytes was seen in irradiated tumours compared to non-irradiated ones, but statistical significance (*p* = 0.3429) was not reached (Fig. 6a). The proportion of migratory monocytes/macrophages increased (*p* = 0.0286), whereas the proportion of macrophages in stage M1 (*p* = 0.0286) and M2 (*p* = 0.1143) decreased in irradiated tumours compared to non-irradiated ones, respectively. In addition, flow cytometry charts of the different subpopulations in non-irradiated and irradiated tumours are shown in Fig. 6b and 6c, respectively. The gating strategy for selecting the subpopulations is shown in Supplementary Fig. 4.

### RT increases [^18^F]F-DPA uptake in FaDu cells

**Fig. 7:**
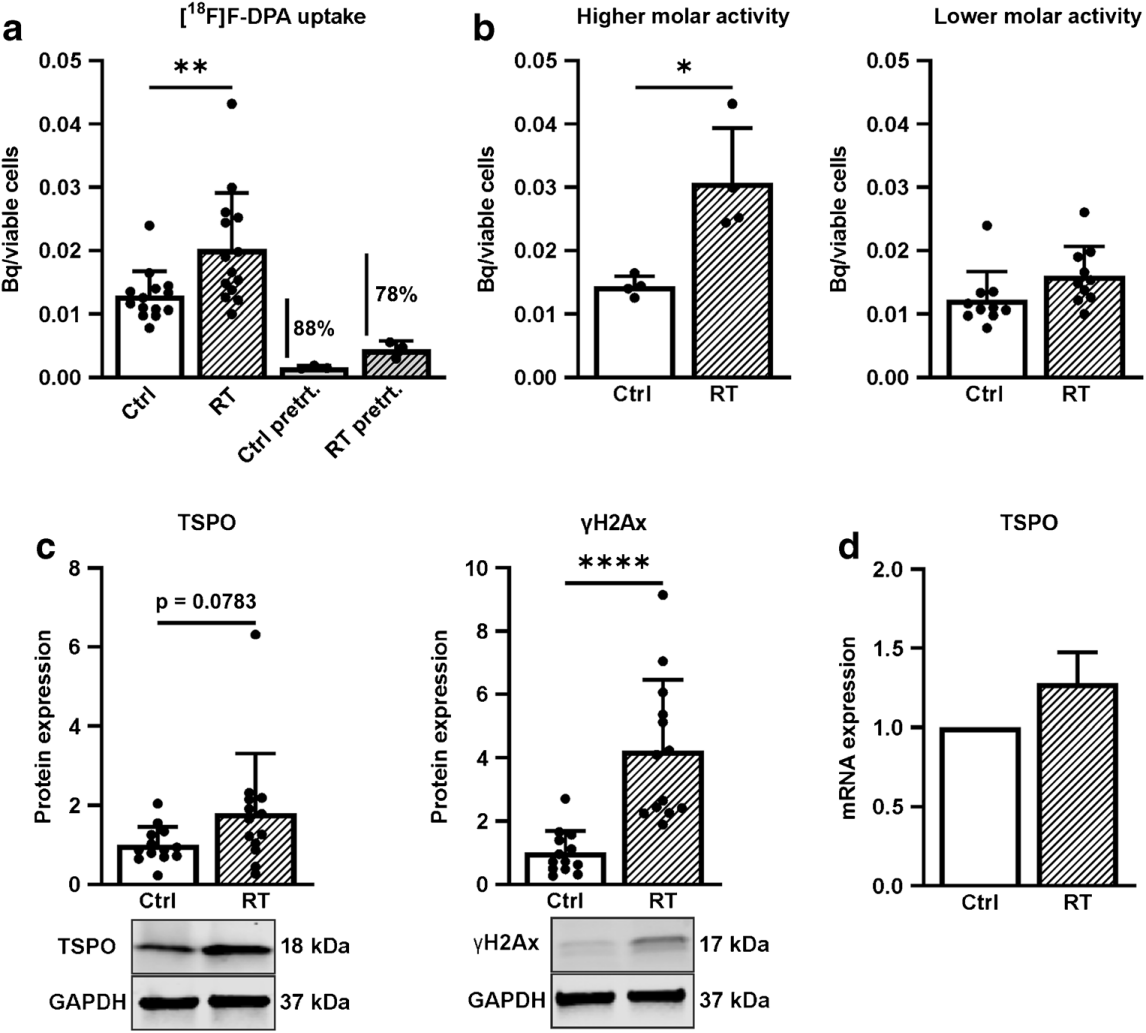
**a** In vitro uptake of [^18^F]F-DPA in non-irradiated (Ctrl), irradiated (RT, 5 × 2 Gy) and PK11195 pre-treated FaDu cells (Pretrt., 10 μM, 30 min prior and during the tracer incubation). Data is expressed as ^18^F-radioactivity in relation to viable cells, mean ± SD, *n* = 14 (Ctrl and RT), *n* = 4 (pre-treated). **b** Data shown in **a** divided into [^18^F]F-DPA uptake with higher (358 GBq/μmol) and lower (10 GBq/μmol) molar activity in Ctrl and RT FaDu cells. **c** Expression of TSPO and γH2Ax (DNA damage marker) proteins in cells shown in **a**. Data is expressed as relative protein expression normalized to housekeeping gene GAPDH, mean ± SD, *n* = 14. **d** TSPO mRNA expression levels in cells shown in **a**. The bars represent TSPO mRNA relative to the average level of housekeeping genes TBP and RPLP0, data is expressed as mean ± SD, *n* = 14. *p < 0.05, **p < 0.01, ***p < 0.001, ****p < 0.0001 are considered to be statistically significant compared to controls, by two-tailed Student’s *t* test. *TSPO* Translocator protein, *TBP* TATA-binding protein, *RPLP0* 60S acidic ribosomal protein P0

Significantly higher (*p* = 0.0087) [^18^F]F-DPA uptake was seen in irradiated cells compared to non-irradiated cells (Fig. 7a). Pre-treatment with PK11195 reduced the uptake by 88% and 78% in non-irradiated and irradiated cells compared to corresponding non-treated cells, respectively. With higher A_m_ the [^18^F]F-DPA uptake increased approximately two-fold after RT compared to non-irradiated cells, whereas only half of that was seen with lower A_m_ (Fig. 7b). An increase, though not significant (*p* = 0.0783), in TSPO protein expression was seen after irradiation in comparison to non-irradiated cells, whereas the mRNA levels did slightly (30%, *p* = 0.2129) increase without reaching significance (Fig. 7c and 7d). Increased (*p* < 0.0001) γH2Ax protein expression in irradiated cells compared to non-irradiated cells confirmed successful RT (Fig. 7c).

### No effect of RT in TSPO siRNA cells

**Fig. 8:**
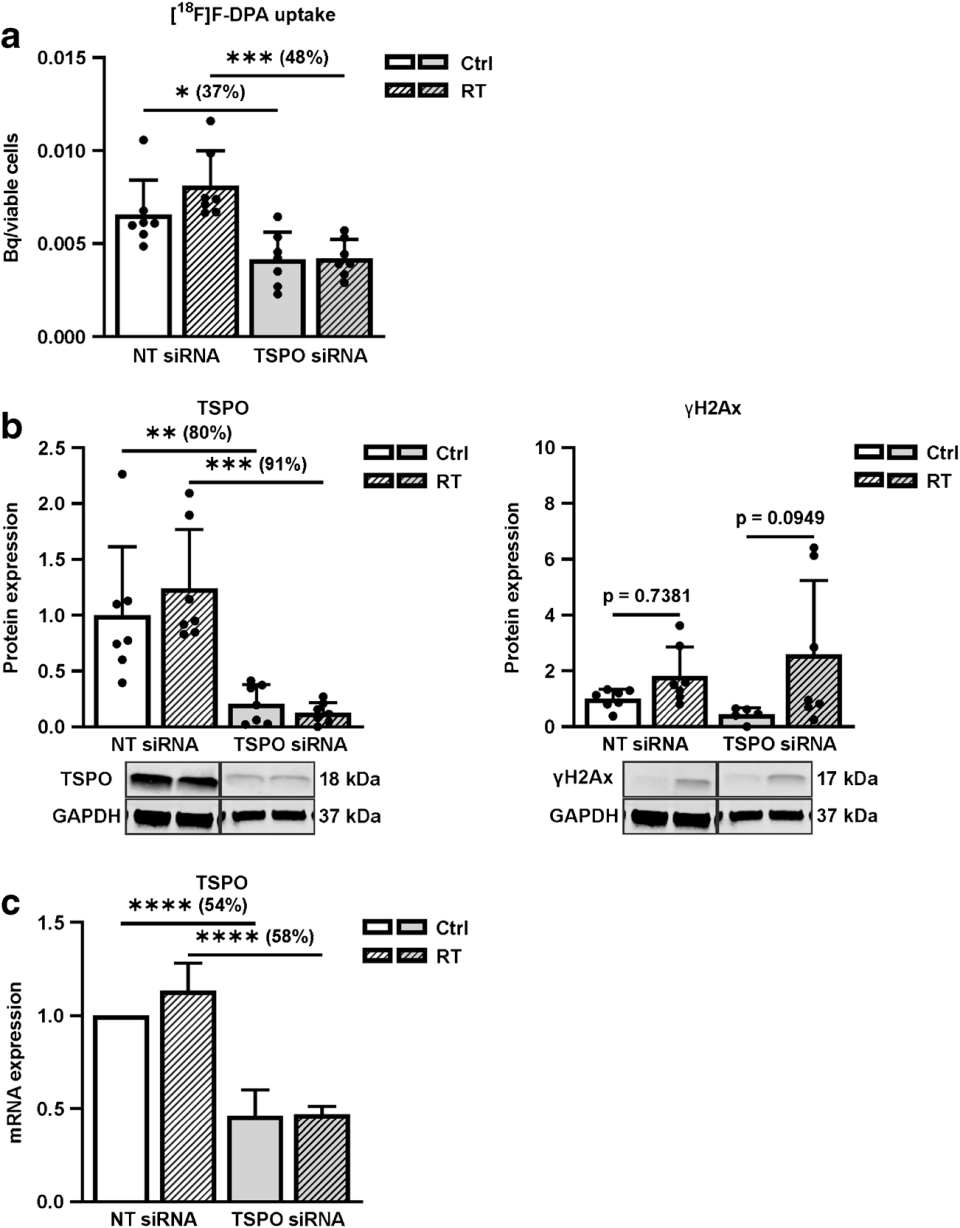
**a** Uptake of [^18^F]F-DPA in non-irradiated (Ctrl) and irradiated (RT, 5 × 2 Gy), non-targeting (NT siRNA) and TSPO silenced (TSPO siRNA), FaDu cells. Data is expressed as ^18^F-radioactivity uptake in relation to viable cells, mean ± SD, *n* = 7 **b** Expression of TSPO and γH2Ax (DNA damage marker) proteins. Data is expressed as relative protein expression normalized to NT siRNA control (GAPDH used as housekeeping gene), mean ± SD, *n* = 7. **c** TSPO mRNA expression was analysed by RT-qPCR. The bars represent TSPO mRNA relative to the average of housekeeping genes TBP and RPLP0, data is expressed as mean ± SD, *n* = 7. *p < 0.05, **p < 0.01, ***p < 0.001, ****p < 0.0001; ^18^F-radioactivity in non-irradiated NT siRNA or TSPO siRNA cells vs irradiated cells, respectively, by two-way ANOVA. *TSPO* Translocator protein, *TBP* TATA-binding protein, *RPLP0* 60S acidic ribosomal protein P0

The uptake of [^18^F]F-DPA in siRNA cells (Fig. 8a) was performed with lower A_m_ (10 GBq/μmol) [^18^F]F-DPA. The uptake decreased by 37% (*p* = 0.0435) in non-irradiated TSPO siRNA cells and by 48% (*p* = 0.0007) in irradiated TSPO siRNA cells compared to respective NT siRNA cells. Irradiation did not increase the tracer uptake in TSPO siRNA cells compared to non-irradiated TSPO siRNA cells (*p* = 0.9998). Even though irradiation did increase the [^18^F]F-DPA uptake in NT siRNA cells compared to non-irradiated NT siRNA cells, significance (*p* = 0.2905) was not reached.

Successful TSPO silencing was confirmed by WB and RT-qPCR analyses. The TSPO protein expression was reduced on average by 80% (*p* = 0.0079) and the mRNA by 54% (*p* < 0.0001) in TSPO siRNA cells compared to NT siRNA cells (Fig. 8b and 8c). Furthermore, neither TSPO protein (*p* = 0.9834) nor mRNA (*p* = 0.9996) levels were affected by RT in TSPO siRNA cells compared to non-irradiated TSPO siRNA cells. An increased, yet not significant, γH2Ax protein expression ensured successful RT-treatment (Fig. 8b).

## Discussion

In order to evaluate the ability of [^18^F]F-DPA to reflect changes in TSPO levels in HNSCC we induced inflammatory responses by RT. In the current study we have shown that RT increases the [^18^F]F-DPA uptake in two different HNSCC xenograft models (FaDu and Cal33), as well as in FaDu cells. Our results are in contrast with a previous study [33], reporting a decreased uptake of [^11^C]DAC in tumour cells and xenografts after carbon ion irradiation (“heavy ion therapy”). We also demonstrate that the RT-induced [^18^F]F-DPA uptake in tumours is long-lasting, at least up to 2 weeks after RT-treatment. The physiological mechanisms regulated by TSPO after RT remain unclear. However, as TSPO has increasingly been suggested to mirror abnormalities in cell metabolism, energy production and oxidative stress [18–20, 22, 34] we speculate that the increased uptake might reflect changes in cell metabolism or ROS homeostasis. Imaging of TSPO in cancer might hence be a useful tool to establish prognosis and indicate response to treatment strategies. Our surprising finding that RT given locally to tumours also resulted in significantly higher [^18^F]F-DPA uptake in the adrenal glands and the kidneys 2 weeks after treatment further supports molecular pathways regulating cell metabolism/energy production to be involved. In fact, since the creation of global TSPO knockout mouse models, several studies have linked TSPO to ATP synthesis and oxygen consumption [13, 35], as well as to fatty acid oxidation, lipid storage, and metabolism [18].

We also compared the [^18^F]F-DPA uptake with that of [^18^F]FDG in FaDu tumours. One week after RT, the average [^18^F]FDG uptake remained unchanged in irradiated compared to non-irradiated tumours, whereas a significantly lower uptake was seen 2 weeks after RT. This is somewhat expected, as the inflammation-induced energy demand will fade over time in relation to cell death in tumours. The fact that the [^18^F]F-DPA uptake did not decrease over time, as was the case with [^18^]FDG, further supports our hypothesis that other than inflammatory factors might be responsible for the increased uptake.

As TSPO is considered to be an inflammatory macrophage marker, we also determined the proportion of monocytes, migratory monocytes/macrophages and macrophages with different polarisation stages (M1 and M2) in the tumours. The proportion of macrophages with polarisation stages of M1 (pro-inflammatory) and M2 (anti-inflammatory) were mainly reduced after RT compared to non-irradiated tumours and cannot therefore be considered the main source for the increased [^18^F]F-DPA uptake. However, RT did increase the proportion of monocytes, which are precursors of macrophages. In a recent study by Narayan et al. [36] the authors demonstrated that monocytes in general express lower TSPO protein and mRNA levels than macrophages. Because less macrophages were detected after RT, our results suggest that [^18^F]F-DPA uptake does not reflect macrophage-induced inflammation. To determine the effect of RT on [^18^F]F-DPA uptake without the influence of the immune system, we used an in vitro approach. Again, our results demonstrate that RT induced [^18^F]F-DPA uptake in FaDu cells.

In order to determine the TSPO-specificity of the tracer uptake we pre-treated tumour bearing mice and cells with the TSPO-selective ligand PK11195. In mice, PK11195 pre-treatment resulted in a significantly higher radioactivity uptake in blood components, naturally a consequence of prevented binding of the tracer to TSPO in tissues, which is compatible with a previous report [37]. PK11195 pre-treatment also increased the radioactivity uptake in the muscle and tumour. The increased uptake may be a consequence of the existing blood pool, and hence an increased amount of unbound tracer, inside these organs. We have previously shown in head and neck cancer patients that the median blood volumes in tumours (5.7 ml/100 g tissue) are rather close to that in the muscle (4.8 ml/100 g tissue) [38]. PK11195 pre-treatment reduced the T/B ratio by 73% and the M/B ratio by 76%. Radiometabolite analyses indeed demonstrated an increased radioactive metabolite profile in tumours after PK11195 pre-treatment compared to non-treated tumours, which was identical to that seen in plasma. In vitro blocking of tumour sections resulted in total blocking of tracer binding. TSPO blocking analyses were also performed with non-irradiated and irradiated FaDu cells in vitro. On average, PK11195 reduced the [^18^F]F-DPA uptake by 88% in non-irradiated cells, whereas the reduction in the tracer uptake was slightly less, 78%, in irradiated cells compared to corresponding non-treated cells.

Finally, we have shown a significantly lower [^18^F]F-DPA uptake in TSPO siRNA FaDu cells. Successful silencing was confirmed by measuring TSPO protein and mRNA levels, which were significantly reduced in TSPO siRNA cells. We found no effect of RT on [^18^F]F-DPA uptake (*p* = 0.9998), TSPO protein (*p* = 0.9834) nor mRNA (*p* = 0.9996) levels in TSPO siRNA cells compared to non-irradiated TSPO siRNA cells, indicating that RT-induced [^18^F]F-DPA uptake is TSPO-dependent.

However, RT did not significantly (*p* = 0.2905) increase [^18^F]F-DPA uptake in NT siRNA cells as one would have expected, which might be due to the lower A_m_ used in those experiments. Another explanation could be a non-specific effect of randomly inserted NT siRNAs in cells. This is also supported by the finding that RT did not significantly increase γH2Ax expression in NT siRNA cells.

TSPO protein expression was also measured from non-irradiated and irradiated FaDu tumours by Western blot and IHC 1 and 2 weeks after RT. Western blots revealed increased, but variable, TSPO protein levels after RT compared to non-irradiated tumours. IHC staining against TSPO revealed no difference in the amount of TSPO-positive cells in non-irradiated and irradiated tumours. The lack of significance and the variability in measured TSPO levels might partly be due to the expression of TSPO in the cell cytosol. Also, the small number of samples analysed in our study might affect the variability. However, irradiation did clearly increase TSPO protein levels in FaDu cells.

Limitations of the current study are small sample number in some experiments. Furthermore, mice were not perfused before removing tissues for ex vivo measurements, which hindered the evaluation of the effect of the blood pool in tumour and muscle after pretreatment with PK11195. As this study was our first attempt to determine the effect of RT on the [^18^F]F-DPA uptake, we did not evaluate aspects related to different RT doses or time periods used for analyses after RT. Unfortunately, we were not able to repeat experiments with high A_m_ [^18^F]F-DPA due to a temporary, but long-lasting, facility closure. Due to this [^18^F]F-DPA was synthesized by two different synthesis pathways, resulting in tracer batches with very different A_m_s. On the other hand, this enabled us to evaluate the effect of the A_m_ on the [^18^F]F-DPA uptake. However, even though final conclusions cannot be drawn from our study, our results with FaDu cells indicate that the A_m_ might affect the uptake of TSPO tracers, also in cancer.

## Conclusion

In the current study, we show that [^18^F]F-DPA can detect changes in TSPO expression after RT in HNSCC. The physiological mechanisms behind this RT-induced uptake need to be further evaluated. Our results suggest that inflammatory factors are not involved. Finally, the pharmacokinetic behaviour of [^18^F]F-DPA in HNSCC indicates this tracer to be suitable for TSPO imaging in cancer.

## Supplementary Information

ESM 1 (DOCX 933 kb).
