# Supplementary material for: Evaluation of [18F]F-DPA as a target for TSPO in head and neck cancer under normal conditions and after radiotherapy
Source: Eur J Nucl Med Mol Imaging. 2020 Dec 19;48(5):1312–26. doi: 10.1007/s00259-020-05115-z (PMC8113193; doi:10.1007/s00259-020-05115-z)
Supplement: Supplementary file 1 — (DOCX 933 kb). [file 259_2020_5115_MOESM1_ESM.docx]

**Supplementary Table 1** Digital IHC analysis of TSPO. Percentages of positively stained cells were analysed using QuPath version 0.2.0-m4. Briefly, colour deconvolution vectors were recorded and cell detection optimized with a training set of images. Automated tissue detection was added to the analysis script, and a set of whole slide images was analysed as a batch. Tissue borders were omitted from the analysis.

| **TSPO** |
| --- |
| **setImageType**('BRIGHTFIELD_H_DAB');  **setColorDeconvolutionStains**('{"Name" : "H-DAB", "Stain 1" : "Hematoxylin", "Values 1" : "0.73096 0.48931 0.47569", "Stain 2" : "DAB", "Values 2" : "0.27188 0.33446 0.90234", "Background" : "255 255 255"}');  **runPlugin**('qupath.imagej.detect.tissue.SimpleTissueDetection2', '{"threshold": 244, "requestedPixelSizeMicrons": 20.0, "minAreaMicrons": 2000000.0, "maxHoleAreaMicrons": 1000000.0, "darkBackground": false, "smoothImage": true, "medianCleanup": true, "dilateBoundaries": false, "smoothCoordinates": true, "excludeOnBoundary": false, "singleAnnotation": true}');  **selectAnnotations**();  **runPlugin**('qupath.lib.plugins.objects.FillAnnotationHolesPlugin', '{}');  **runPlugin**('qupath.imagej.detect.cells.PositiveCellDetection', '{"detectionImageBrightfield": "Hematoxylin OD", "requestedPixelSizeMicrons": 0.5, "backgroundRadiusMicrons": 10.0, "medianRadiusMicrons": 1.0, "sigmaMicrons": 2.0, "minAreaMicrons": 10.0, "maxAreaMicrons": 400.0, "threshold": 0.05, "maxBackground": 2.0, "watershedPostProcess": true, "excludeDAB": true, "cellExpansionMicrons": 5.0, "includeNuclei": true, "smoothBoundaries": true, "makeMeasurements": true, "thresholdCompartment": "Cytoplasm: DAB OD mean", "thresholdPositive1": 0.2, "thresholdPositive2": 0.4, "thresholdPositive3": 0.6000000000000001, "singleThreshold": true}');  **setImageType**('BRIGHTFIELD_H_DAB');  **setColorDeconvolutionStains**('{"Name" : "H-DAB-PPH3", "Stain 1" : "Hematoxylin", "Values 1" : "0.63063 0.71426 0.30355 ", "Stain 2" : "DAB", "Values 2" : "0.2474 0.51166 0.8228", "Background" : " 255 255 255 "}');  **runPlugin**('qupath.imagej.detect.tissue.SimpleTissueDetection2', '{"threshold": 244, "requestedPixelSizeMicrons": 20.0, "minAreaMicrons": 2000000.0, "maxHoleAreaMicrons": 1000000.0, "darkBackground": false, "smoothImage": true, "medianCleanup": true, "dilateBoundaries": false, "smoothCoordinates": true, "excludeOnBoundary": false, "singleAnnotation": true}');  **selectAnnotations**();  **runPlugin**('qupath.lib.plugins.objects.FillAnnotationHolesPlugin', '{}');  **runPlugin**('qupath.imagej.detect.cells.PositiveCellDetection', '{"detectionImageBrightfield": "Optical density sum", "requestedPixelSizeMicrons": 1.0, "backgroundRadiusMicrons": 8.0, "medianRadiusMicrons": 2.0, "sigmaMicrons": 3.0, "minAreaMicrons": 10.0, "maxAreaMicrons": 400.0, "threshold": 0.0, "maxBackground": 2.0, "watershedPostProcess": true, "excludeDAB": false, "cellExpansionMicrons": 5.0, "includeNuclei": true, "smoothBoundaries": true, "makeMeasurements": true, "thresholdCompartment": "Nucleus: DAB OD mean", "thresholdPositive1": 0.2, "thresholdPositive2": 0.4, "thresholdPositive3": 0.6, "singleThreshold": true}'); |

**Supplementary Table 2** Digital IHC analysis of active caspase-3 and PHH3. Percentages of positively stained cells were analysed using QuPath version 0.2.0-m2. Briefly, colour deconvolution vectors were recorded and cell detection optimized with a training set of images. Automated tissue detection was added to the analysis script, and a set of whole slide images was analysed as a batch. Most positively-stained areas were selected visually and a circular region of interest (ROI) with a 700-µm radius drawn and the percentages of positive cells recorded. The radius was chosen in order to capture comparative areas from all-sized and differently shaped tissues. Tissue borders were omitted from the analysis.

| **Cleaved Caspase-3 and PHH3** |
| --- |
| **setImageType**('BRIGHTFIELD_H_DAB');  **setColorDeconvolutionStains**('{"Name" : "H-DAB default", "Stain 1" : "Hematoxylin", "Values 1" : "0.65111 0.70119 0.29049 ", "Stain 2" : "DAB", "Values 2" : "0.26917 0.56824 0.77759", "Background" : " 255 255 255 "}');  **runPlugin**('qupath.imagej.detect.tissue.SimpleTissueDetection2', '{"threshold": 244, "requestedPixelSizeMicrons": 20.0, "minAreaMicrons": 2000000.0, "maxHoleAreaMicrons": 1000000.0, "darkBackground": false, "smoothImage": true, "medianCleanup": true, "dilateBoundaries": false, "smoothCoordinates": true, "excludeOnBoundary": false, "singleAnnotation": true}');  **selectAnnotations**();  **runPlugin**('qupath.lib.plugins.objects.FillAnnotationHolesPlugin', '{}');  **runPlugin**('qupath.imagej.detect.cells.PositiveCellDetection', '{"detectionImageBrightfield": "Optical density sum", "requestedPixelSizeMicrons": 0.5, "backgroundRadiusMicrons": 7.0, "medianRadiusMicrons": 1.0, "sigmaMicrons": 1.5, "minAreaMicrons": 10.0, "maxAreaMicrons": 400.0, "threshold": 0.05, "maxBackground": 2.0, "watershedPostProcess": true, "excludeDAB": false, "cellExpansionMicrons": 5.0, "includeNuclei": true, "smoothBoundaries": true, "makeMeasurements": true, "thresholdCompartment": "Nucleus: DAB OD mean", "thresholdPositive1": 0.12, "thresholdPositive2": 0.4, "thresholdPositive3": 0.6, "singleThreshold": true}'); |

**
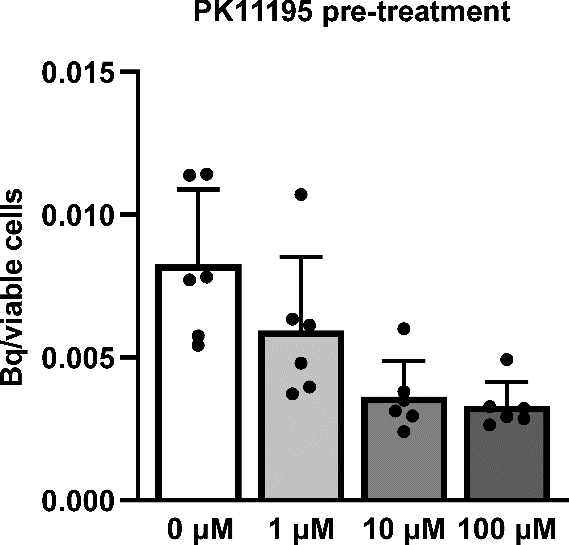
**

**Supplementary Fig. 1** Effect of different concentrations (1 µM, 10 µM, and 100 µM) of PK11195 pre-treatment on *in vitro* [^18^F]F-DPA uptake in FaDu cells.

**
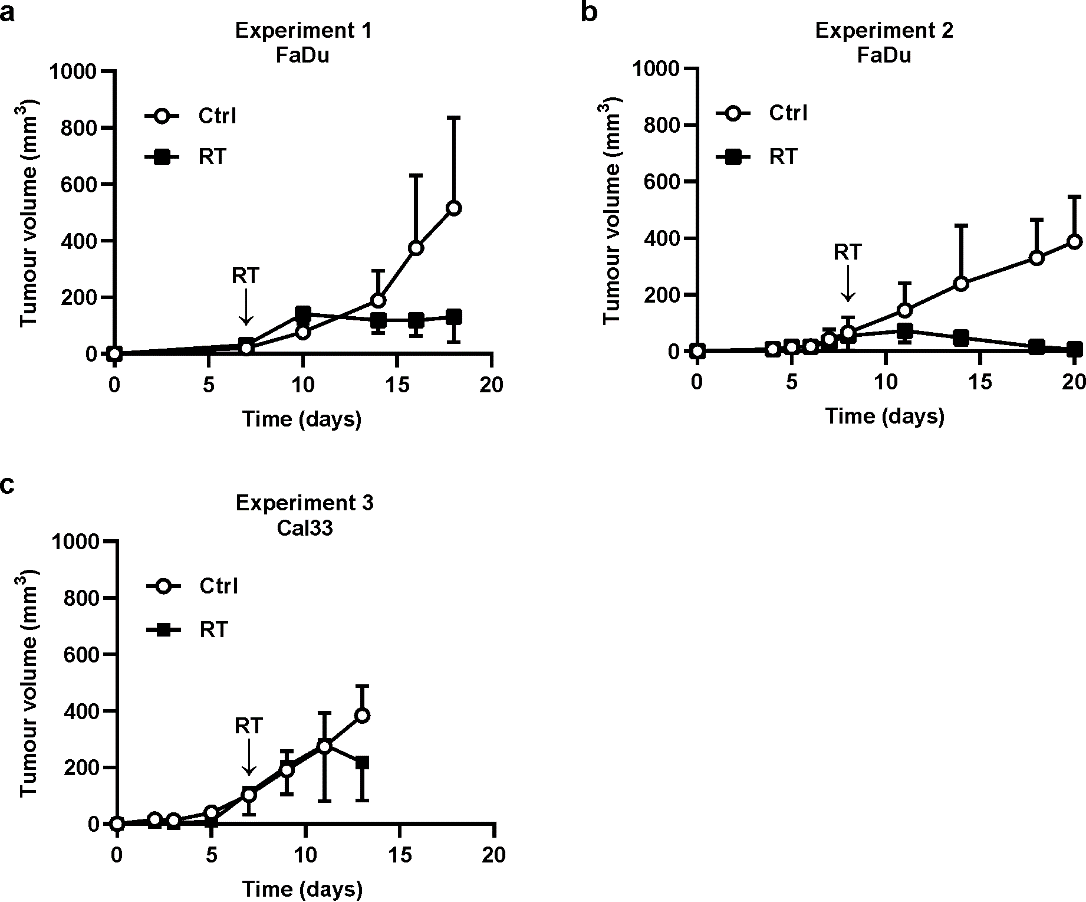
**

**Supplementary Fig. 2** Tumour growth curves of FaDu xenografts in (**a**) experiment 1 and (**b**) experiment 2, and (**c**) Cal33 xenografts in experiment 3.

**Supplementary Table 3** Measured ^18^F-radioactivity at 40 min after injection of [^18^F]F-DPA in non-irradiated (Ctrl) and FaDu tumour bearing mice irradiated locally to tumour (RT, 2 × 5 Gy). Data is expressed as percentage of injected dose per gram tissue (% ID/g), mean ± SD, n = 3/group. *LI* large intestine, *SI* small intestine, *subcut.* subcutaneous, *BAT* brown adipose tissue.

|  | **Ctrl**  **% ID/g tissue** | | **RT**  **% ID/g tissue** | |
| --- | --- | --- | --- | --- |
| **Tissue** | **Mean** | **SD** | **Mean** | **SD** |
| **Blood** | 0.48 | 0.02 | 0.52 | 0.01 |
| **Plasma** | 0.22 | 0.12 | 0.18 | 0.07 |
| **Erythrocytes** | 0.72 | 0.09 | 0.81 | 0.09 |
| **Heart** | 9.43 | 3.38 | 11.01 | 0.94 |
| **Lungs** | 22.57 | 4.53 | 24.91 | 2.89 |
| **Liver** | 13.50 | 0.51 | 17.36 | 3.48 |
| **Adrenal glands** | 64.30 | 23.34 | 112.51 | 8.00 |
| **Kidneys** | 25.06 | 1.28 | 41.18 | 7.62 |
| **Spleen** | 18.25 | 4.29 | 18.60 | 1.82 |
| **Pancreas** | 6.38 | 0.88 | 9.41 | 2.60 |
| **Gallbladder** | 10.98 | 2.61 | 12.02 | 4.09 |
| **Stomach** | 4.53 | 2.20 | 4.13 | 1.99 |
| **SI** | 9.75 | 0.27 | 12.98 | 3.13 |
| **LI** | 5.81 | 0.63 | 7.76 | 1.78 |
| **Ovaries** | 8.05 | 1.46 | 8.13 | 2.37 |
| **Fat (subcut.)** | 4.02 | 1.78 | 9.45 | 4.75 |
| **Fat (BAT)** | 56.60 | 35.91 | 20.53 | 21.70 |
| **Muscle** | 1.39 | 0.81 | 1.83 | 0.60 |
| **Thymus** | 38.74 | 17.94 | 26.16 | 15.09 |
| **Salivary glands** | 10.30 | 6.00 | 9.86 | 4.24 |
| **Brain** | 0.74 | 0.14 | 0.94 | 0.13 |
| **Bone** | 1.82 | 0.60 | 2.49 | 0.92 |
| **Urine** | 4.22 | 1.68 | 5.97 | 2.64 |


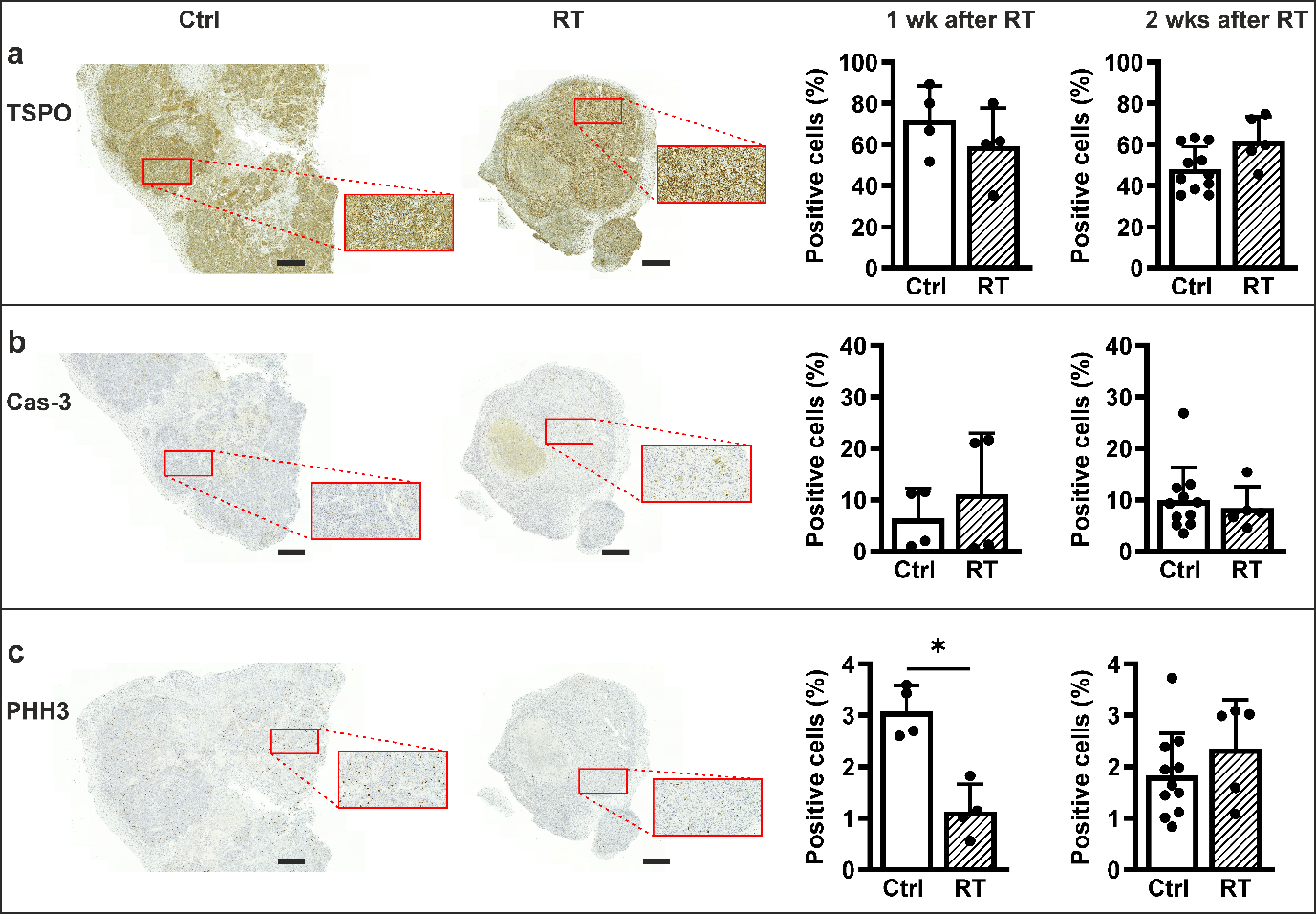


**Supplementary Fig. 3** Immunohistochemical images representing staining against (**a**) TSPO, (**b**) apoptosis (Cas-3) and (**c**) proliferation (PHH3) in non-irradiated (Ctrl) and irradiated (RT) tumours. No change was seen in the percentage of TSPO and Cas-3 positive cells after RT. A significant decrease in the number of proliferative cells was detected one week after RT, whereas this difference was diminished two weeks after RT. Values are percentage of positive cells, mean ± SD (1 week; n = 4/group, 2 weeks; n = 11 for Ctrl and n = 5 for RT). *p < 0.05 is considered to be statistically significant compared to controls, by two-tailed Student’s *t*-test.

**
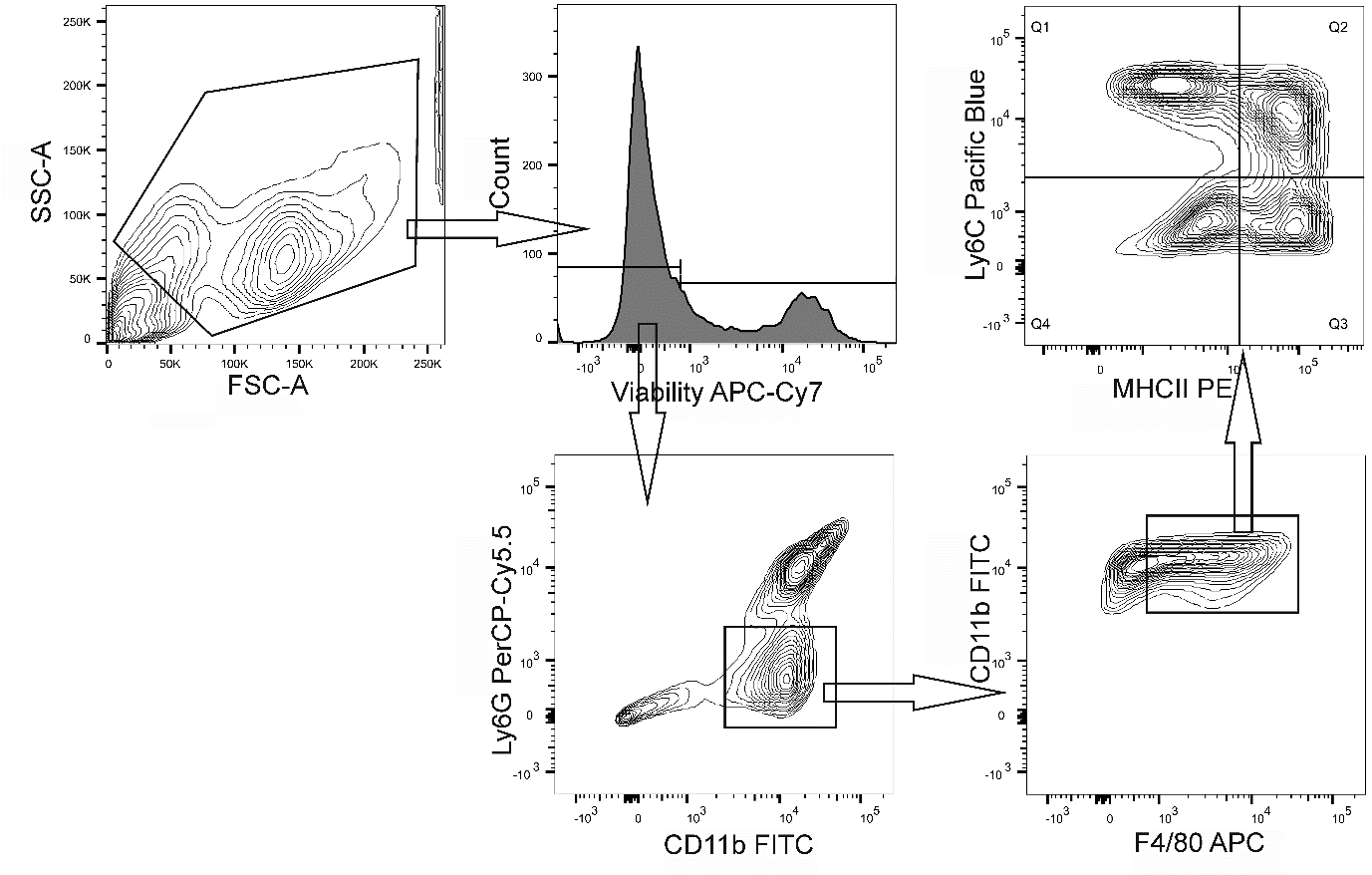
**

**Supplementary Fig. 4** Gating strategy for selecting the monocytes/macrophages. Cells were first gated according to their size and granularity (FSC and SSC, respectively) before selecting cells that were negative for the viability marker and therefore alive. From this population, CD11b-positive myeloid cells were selected while excluding Ly6G-positive neutrophils. In the next step, only F4/80- positive monocytes/macrophages were selected and this population was then finally divided according to MHC class II and Ly6C expression into four different populations, namely Ly6C+ MHCII- inflammatory monocytes (Q1), Ly6C+ MHCII+ migratory/immature macrophages (Q2), Ly6C- MHCII+ M1 tumour-associated macrophages (Q3) and Ly6C- MHCII- M2 tumour-associated macrophages (Q4).
